# Supplementary material for: Extracting Informative Glycan-Specific Ions From Glycopeptide MS/MS Spectra With GlyCounter
Source: Mol Cell Proteomics. 2025 Oct 15;24(12):101085. doi: 10.1016/j.mcpro.2025.101085 (PMC12701960; doi:10.1016/j.mcpro.2025.101085)
Supplement: Supplymentry Figures [file mmc5.pdf]

Supplemental Information

Extracting informative glycan-specific ions from glycopeptide MS/MS spectra with GlyCounter

Kathryn Kothlow[1], Haley M. Schramm[1], Kayla A. Markuson[1], Jacob H. Russell[1], Emmajay Sutherland[1], Tim S. Veth[1], Ruby Zhang[1], Anna G. Duboff[1], Vishnu R. Tejus[1], Leah E. McDermott[1], Laura S. Dräger[1¥], and Nicholas M. Riley[1*]

[1]Department of Chemistry, University of Washington, Seattle, WA, 98195

*address correspondence to: nmriley@uw.edu

Supplemental Materials and Methods

Supplemental Figures

Supplementary Figure 1. Total ion current (TIC) fraction from B-type ions in HCD spectra for N-glycopeptides.
Supplementary Figure 2. Total ion current (TIC) fraction from B-type ions in EThcD spectra for N-glycopeptides.
Supplementary Figure 3. Total ion current (TIC) fraction from B-type ions in EThcD spectra for O-glycopeptides.
Supplementary Figure 4. The number of non-glycopeptide spectra included as LikelyGlyco based on required numbers of oxonium ions.
Supplementary Figure 5. Peak depth thresholds for oxonium ions in HCD spectra.
Supplementary Figure 6. Peak depth thresholds for oxonium ions in EThcD spectra.
Supplementary Figure 7. Results for species that are not glycosylated.
Supplementary Figure 8. Search time comparisons.
Supplementary Figure 9. The Ynaught interface.
Supplementary Figure 10. Categorization of glycopeptides remaining after PNGaseF treatment.
Supplementary Figure 11. Adapting to non-ideal m/z scan ranges.
References.

Supplemental Files (in separate Excel files)

Supplementary File 1. Example GlyCounter output files.
Supplementary File 2. GlyCounter guide and tutorial.
Supplementary File 3. Example GlyCounter custom ion upload file.
Supplementary File 4. Data tables used for creating figures.

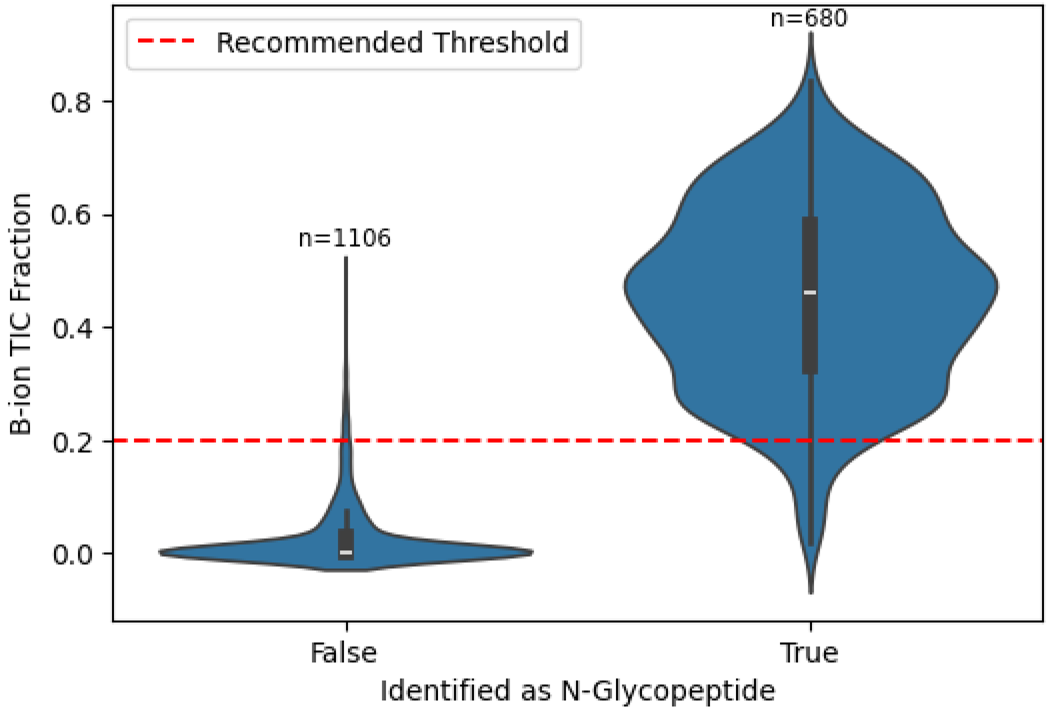

Supplementary Figure 1. Total ion current (TIC) fraction from B-type ions in HCD spectra for N-glycopeptides. Data from PXD023448 compares the fraction of total ion current that can be attributed to glycan-specific B-type ions for spectra identified as nonmodified peptides (labeled as false on the x-axis) and spectra identified as N-glycopeptides (labeled as true on the x-axis). The dashed horizontal line shows a TIC fraction of 0.20, the default threshold for GlyCounter for HCD spectra.

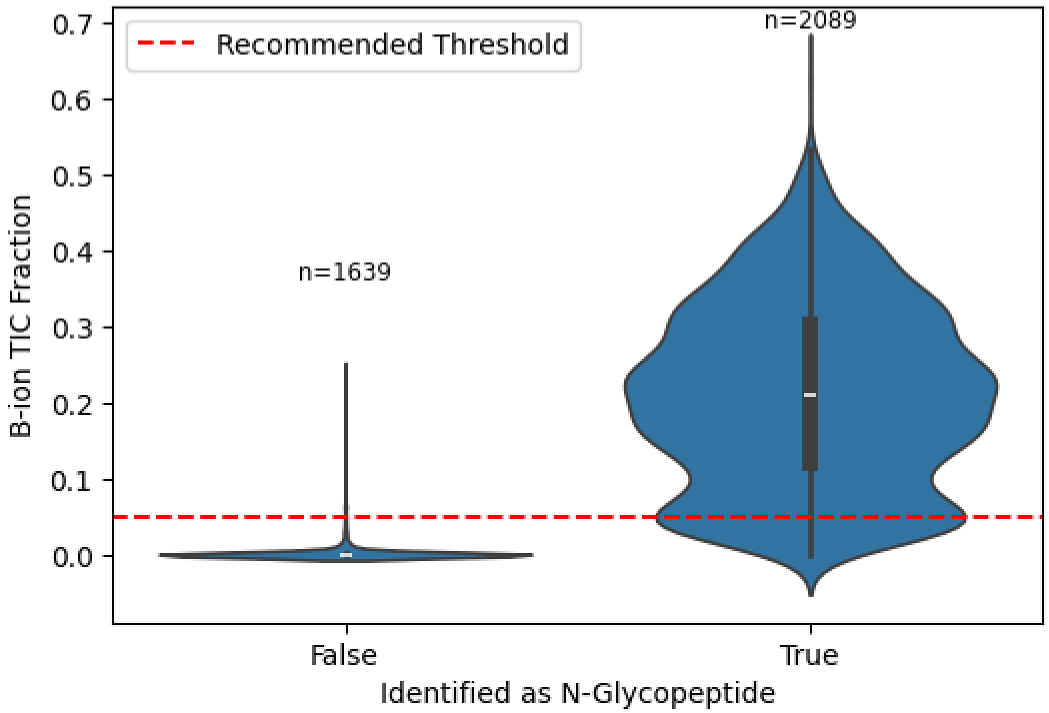

Supplementary Figure 2. Total ion current (TIC) fraction from B-type ions in EThcD spectra for N-glycopeptides. Data from PXD058153 compares the fraction of total ion current that can be attributed to glycan-specific B-type ions for spectra identified as nonmodified peptides (labeled as false on the x-axis) and spectra identified as N-glycopeptides (labeled as true on the x-axis). The dashed horizontal line shows a TIC fraction of 0.05, the default threshold for GlyCounter for ETD-based spectra, which includes EThcD.

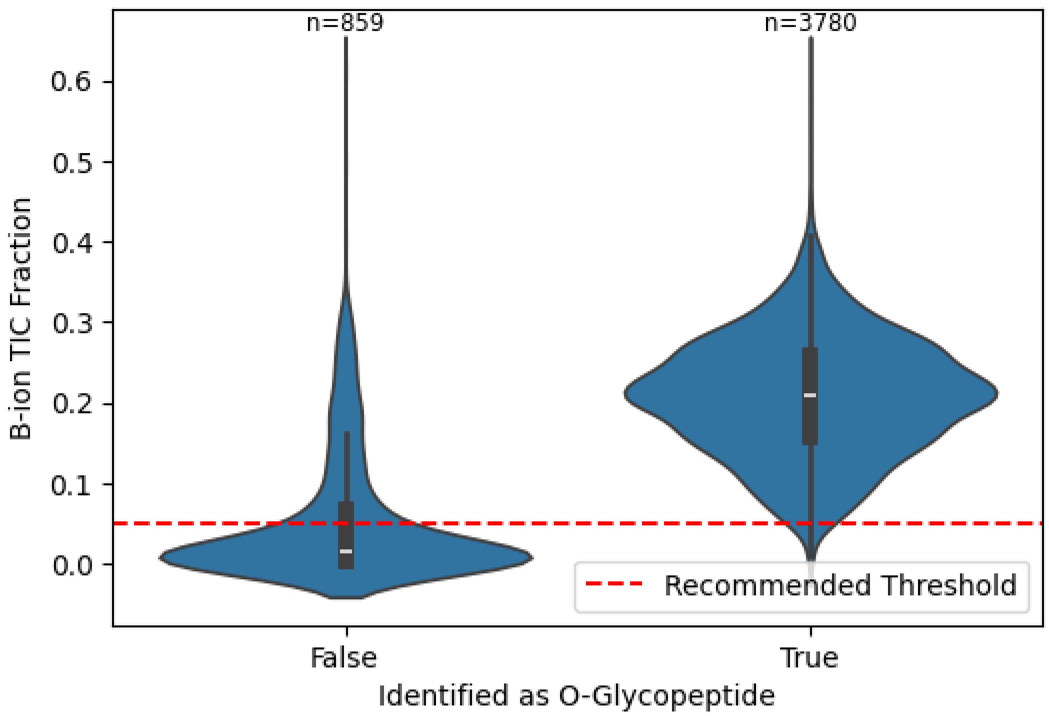

Supplementary Figure 3. Total ion current (TIC) fraction from B-type ions in EThcD spectra for O-glycopeptides. Data from PXD058153 compares the fraction of total ion current that can be attributed to glycan-specific B-type ions for spectra identified as nonmodified peptides (labeled as false on the x-axis) and spectra identified as O-glycopeptides (labeled as true on the x-axis). The dashed horizontal line shows a TIC fraction of 0.05, the default threshold for GlyCounter for ETD-based spectra, which includes EThcD.

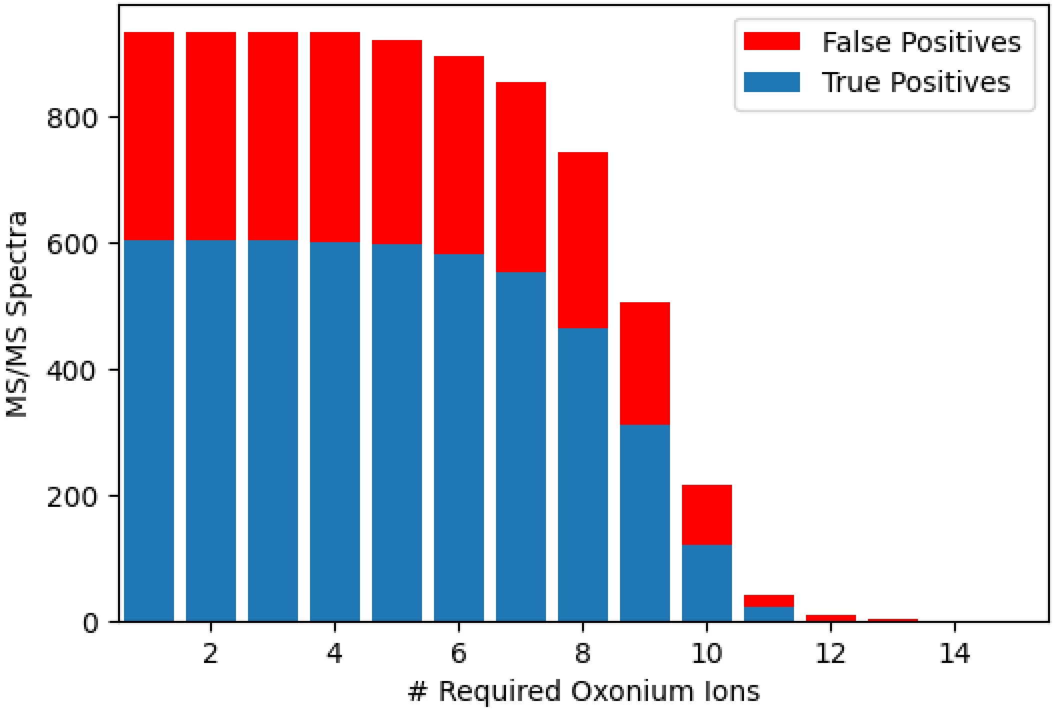

Supplementary Figure 4. The number of non-glycopeptide spectra included as LikelyGlyco based on required numbers of oxonium ions. Data from PXD023448 compares the number of false positives (red) for the LikelyGlyco calculation, i.e., the number of nonmodified peptides, as determined by a database search, that are marked as LikelyGlyco, when only requiring the specified number of oxonium ions (x-axis). The graph also shows the number of true positives (blue), i.e., the number of N-glycopeptides identified by a database search that are marked as LikelyGlyco, when requiring the specified number of oxonium ions. (The default setting for GlyCounter when selecting more than 15 oxonium ions is 8 required ions to balance sensitivity and selectivity).

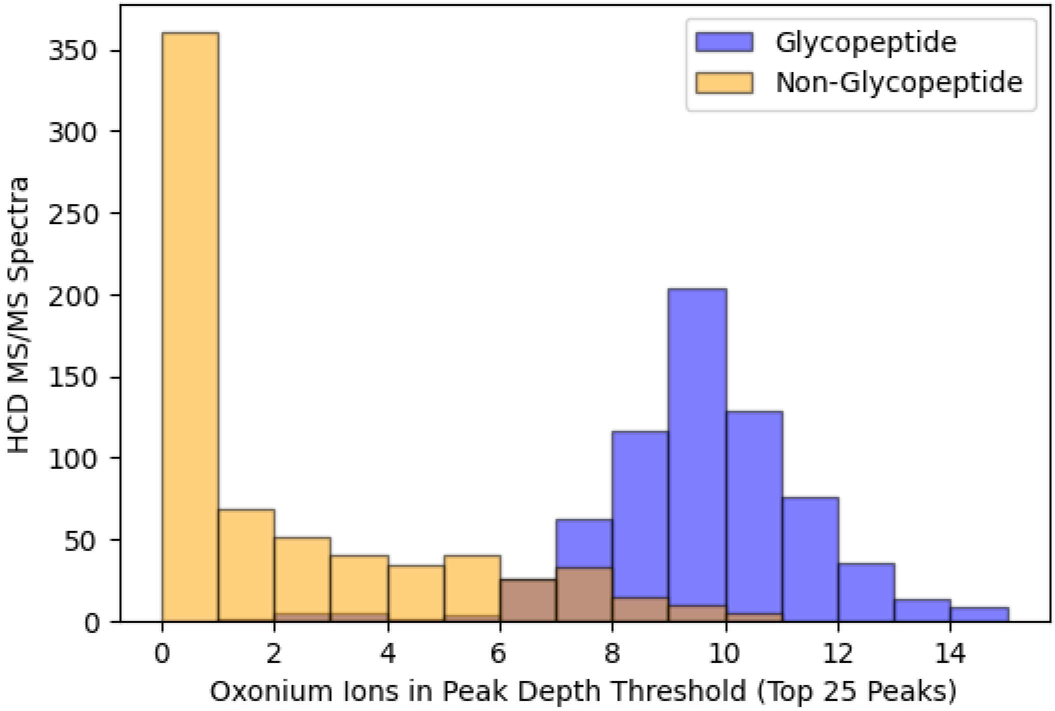

Supplementary Figure 5. Peak depth thresholds for oxonium ions in HCD spectra. Data from PXD023448 compares the number of spectra with a specified number of oxonium ions in the top 25 peaks of HCD spectra of nonmodified peptides (gold) and N-glycopeptides (purple), as assigned by a database search. The top 25 peak depth threshold is the default setting for HCD spectra in GlyCounter.

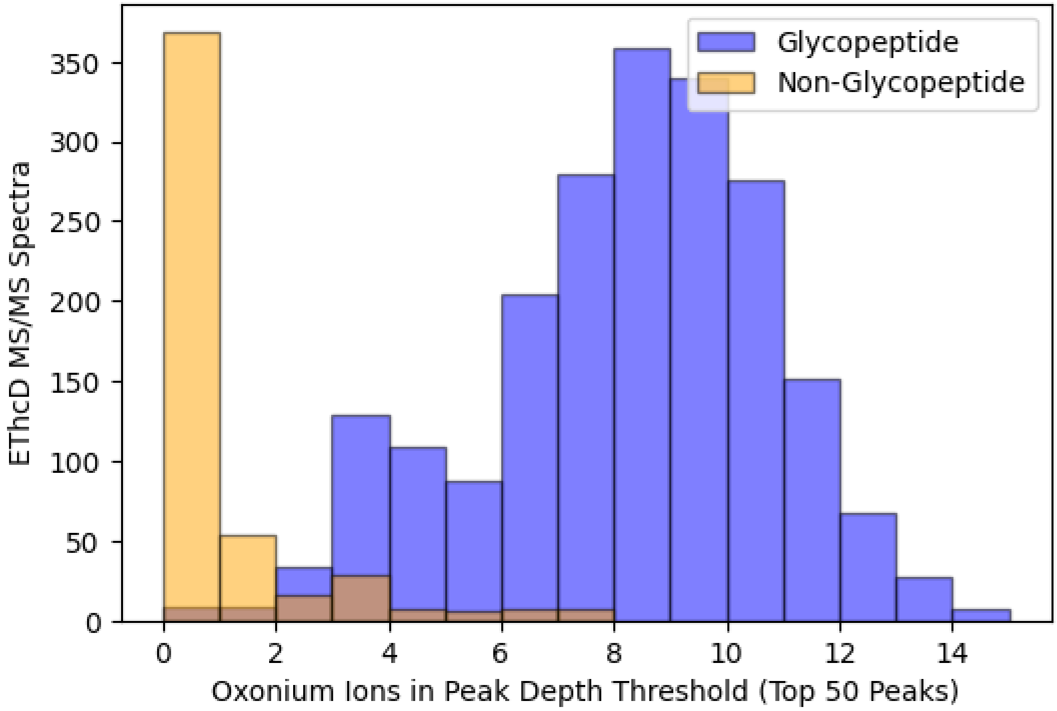

Supplementary Figure 6. Peak depth thresholds for oxonium ions in EThcD spectra. Data from PXD058153 compares the number of spectra with a specified number of oxonium ions in the top 50 peaks of EThcD spectra of nonmodified peptides (gold) and N-glycopeptides (purple), as assigned by a database search. The top 50 peak depth threshold is the default setting for ETD spectra in GlyCounter, which includes EThcD spectra.

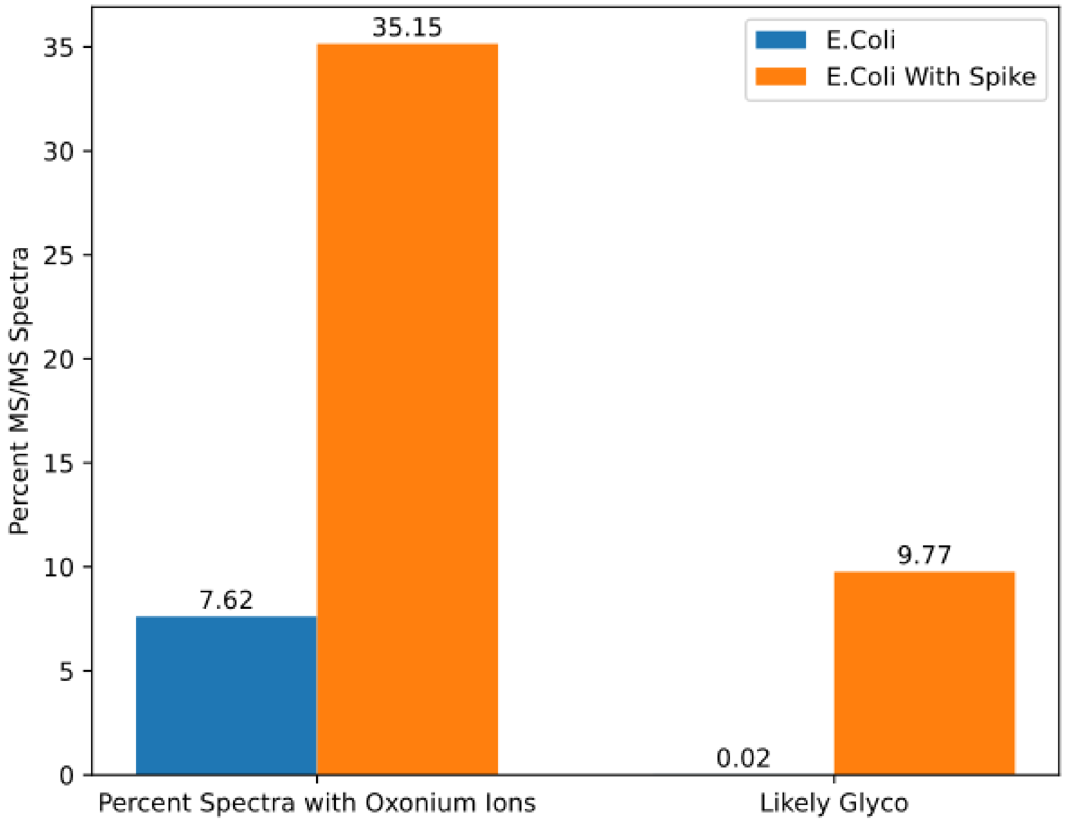

Supplementary Figure 7. Results for species that are not glycosylated. Proteomics data from an E. coli sample (no native glycosylation) was compared to an *E. coli* sample with 5 glycoproteins (CD80, CD4, alpha-1-acid glycoprotein, bovine fetuin, and the SARS CoV-2 spike protein) spiked in. The percentage of MS/MS spectra containing oxonium ions according to GlyCounter were compared, showing that dramatically fewer native *E. coli* spectra contained oxonium ions compared to the glycoprotein spiked sample. GlyCounter's LikelyGlyco spectrum assignment was also compared between the two tests, which resulted in a markedly higher percentage of spectra being identified as LikelyGlyco for the spiked sample when compared to the effectively zero LikelyGlyco assignments in the native *E. coli* data. Overall, we expect that there may be some minimal error in GlyCounter's identification of ions due to noise, but we do not expect this to significantly impact analysis.

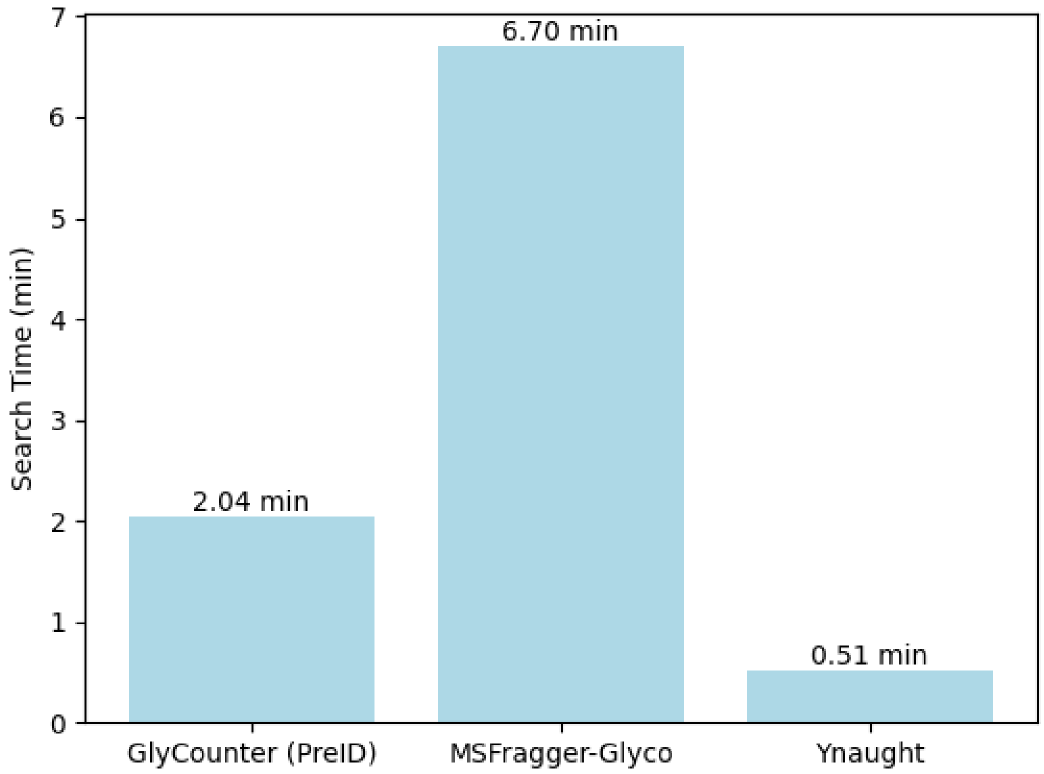

Supplementary Figure 8. Search time comparisons. We tested the search time of GlyCounter using a raw file from the mouse brain data set of Liu et al.[1] All searches were run on the same computer. All GlyCounter settings were left as default and all ions were checked. MSFraggerGlyco was run through FragPipe using the N-glyco-HCD workflow with 32 GB of RAM and parallelism set to 16. The mouse proteome was downloaded from UniProt and the provided Mouse N-glycans-182 database was used for searching. Other settings remained unchanged. Ynaught was then run with the psm.tsv file from FragPipe and the same glycan database as used in the MSFragger-Glyco search. All Y-ions were checked and other settings were left as default.

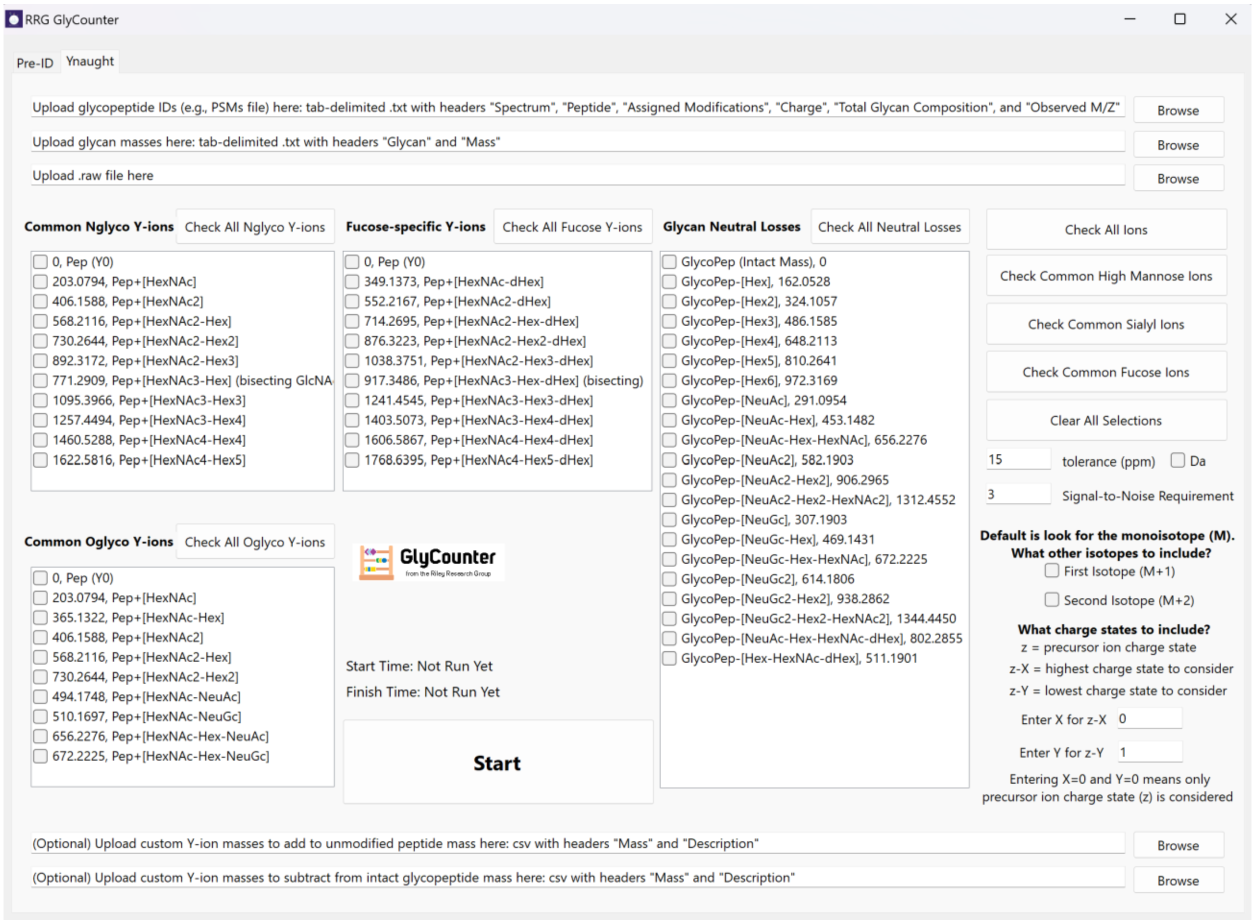

Supplementary Figure 9. The Ynaught interface. Ynaught requires 3 file inputs including the identification file from a database search (formatted according to the example on GitHub), a glycan masses list, and the .raw data file. Ynaught currently does not support .mzML files. Users select which Y-ions (peptide mass + glycan mass) or neutral losses (glycopeptide mass – glycan mass) to search for. Settings include ppm tolerance and signal-to-noise requirements. Ynaught is able to look for multiple isotopes and charge states depending on user inputs. Optional uploads allow for either custom Y-ion masses to add to the precursor peptide mass or custom neutral losses to subtract from the precursor glycopeptide mass.

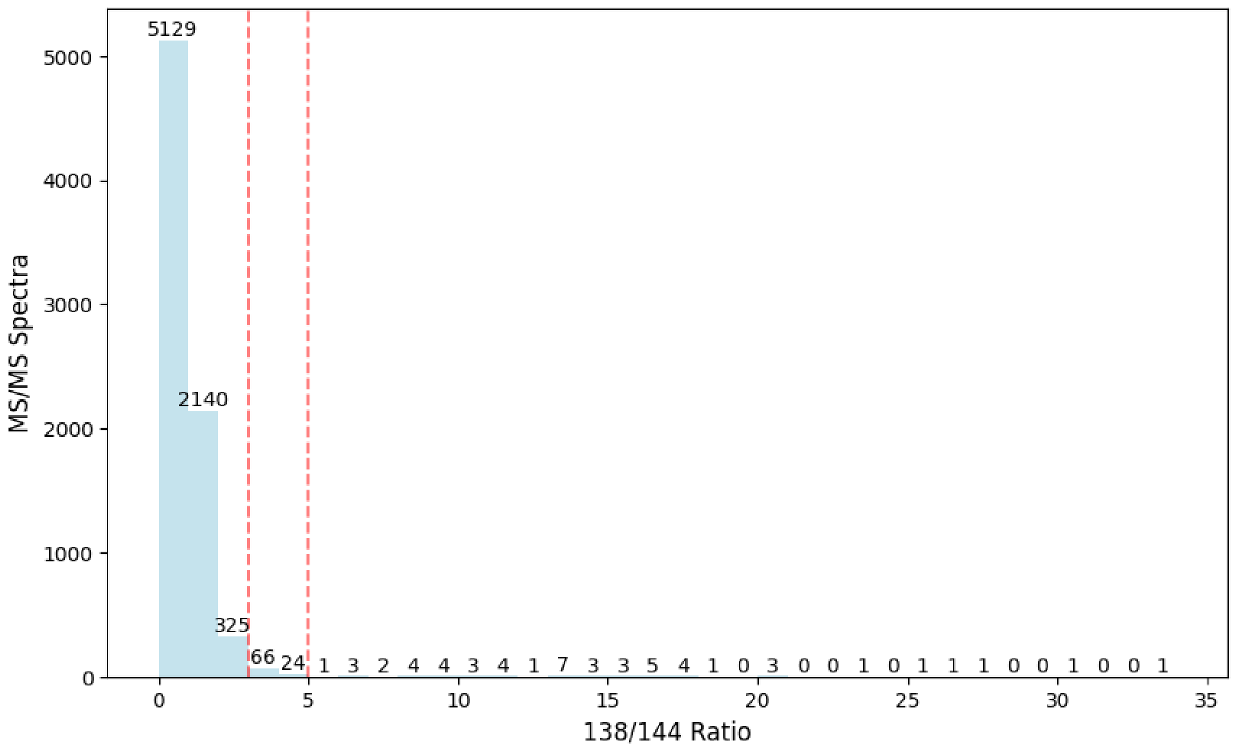

Supplementary Figure 10. Categorization of glycopeptides remaining after PNGaseF treatment. The 138/144 ratio has been shown to distinguish N-linked and O-linked glycopeptides. Generally, O-glycopeptides have a 138/144 ratio of 3 or less, and N-glycopeptides have a 138/144 ratio of 5 or greater.[2] Using GlyCounter, we calculated the 138/144 ratios of spectra from a deglycoproteomics experiment. A large majority of spectra fall into the O-glycopeptide category.

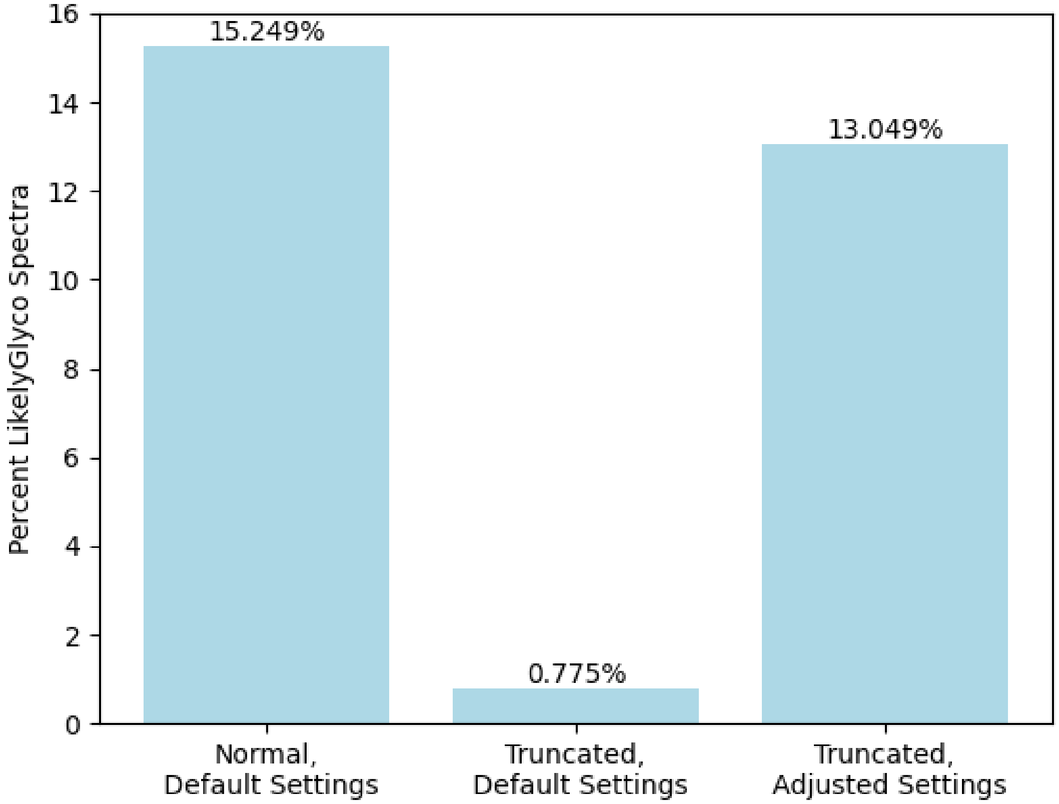

Supplementary Figure 11. Adapting to non-ideal m/z scan ranges. To demonstrate the effect on a spectrum being considered "LikelyGlyco" of experiments with scan ranges that exclude some oxonium ions, we used a spectral file from Batth et al.[3] that originally had an MS/MS scan range beginning at 100 m/z (Normal). We then truncated the file so that all peaks below 190 m/z would not be included in the file (Truncated). Finally, we tested GlyCounter on both files. With default settings, the oxonium ions missed by the truncated scan range cause the percentage of likely glycopeptide spectra to dramatically drop. However, by adjusting the settings to only require 4 oxonium ions in the top 25 peaks instead of 8, we see that the percentage of likely glycopeptide spectra is similar to the original file.

References

(1) Liu, M.-Q.; Zeng, W.-F.; Fang, P.; Cao, W.-Q.; Liu, C.; Yan, G.-Q.; Zhang, Y.; Peng, C.; Wu, J.-Q.; Zhang, X.-J.; Tu, H.-J.; Chi, H.; Sun, R.-X.; Cao, Y.; Dong, M.-Q.; Jiang, B.-Y.; Huang, J.-M.; Shen, H.-L.; Wong, C. C. L.; He, S.-M.; Yang, P.-Y. pGlyco 2.0 Enables Precision N-Glycoproteomics with Comprehensive Quality Control and One-Step Mass Spectrometry for Intact Glycopeptide Identification. *Nat. Commun.* **2017**, *8* (1), 438. https://doi.org/10.1038/s41467-017-00535-2.

(2) Riley, N. M.; Malaker, S. A.; Driessen, M. D.; Bertozzi, C. R. Optimal Dissociation Methods Differ for N- and O-Glycopeptides. *J. Proteome Res.* **2020**, *19* (8), 3286–3301. https://doi.org/10.1021/acs.jproteome.0c00218.

(3) Batth, T. S.; Francavilla, C.; Olsen, J. V. Off-Line High-pH Reversed-Phase Fractionation for In-Depth Phosphoproteomics. *J. Proteome Res.* **2014**, *13* (12), 6176–6186. https://doi.org/10.1021/pr500893m.
